# A Novel Case

**Published:** 1881-10

**Authors:** 


### EDITORIAL.

### A NOVEL CASE.

Mr. L. B., sixty-three years of age, had during the last fifteen or twenty years lost, by various means, nearly all of the teeth of the upper jaw; having for the last five years, remaining only the two molars of the left side, the left lateral incisor, the right central and the right cuspid. For about five years he has worn a gold plate to which are attached substitutes for all the lost teeth, except the third molar on the right side.

The five remaining natural teeth, were in comparatively good condition till about six months ago, when the lateral incisor began to loosen, and rapidly became more and more loose, till to-day, he applied for treatment or removal. Upon examination, the root was found to have no periostial attachment; about two-thirds of the root was dissolved off, the remaining part was simply sitting or hanging in a cup-shaped socket in the gum, without any attachment whatever to the walls of the socket. The means of attachment, or rather suspension, was not understood, till upon taking hold upon the crown and making a little traction, the tooth slipped off of the pulp, which retained its attachment at the bottom of the socket, and made some resistance to separation from the tooth, till the latter was drawn entirely off. Some pain was experienced in this separation.

After the removal of the tooth, the pulp stood erectly from the bottom of the socket. It was then manipulated to determine its character; it was quite painful upon being extracted or handled, as much so as an ordinary healthy tooth is. Its consistence was that of the ordinary tooth-pulp, and the appearance when torn asunder, was about the same; its attachment to the soft parts was perhaps a little larger than that of the ordinary tooth-pulp just beyond the foramen at the end of the root; but in no other respect did it differ from an ordinary tooth-pulp.

The tenacity of life, and the maintainance of its good condition, was to me a matter of great surprise. How, and why it was, that the agents that dissolved away the root so largely, even to the destruction of its attachment, did not produce disease in, and the death of this pulp is, to say the least, remarkable; and shows that in some cases, at least, this organ which is generally esteemed so very delicate and frail, that even a contact by a foreign body is fraught with danger of disease and perhaps death, is, in some cases at least, endowed with strong vitality and resistance.

The inference must not be drawn from this case that pulps generally have a similar tenacity of life, and resistance to unfavorable surroundings; but that this organ possesses a much greater power of resistance to irritants than is usually supposed is doubtless true. From this case an encouraging lesson may be learned; one that should be used to some purpose in the treatment of exposed pulps.

